# STV-SC: Segmentation and Temporal Verification Enhanced Scan Context for Place Recognition in Unstructured Environment

**DOI:** 10.3390/s22228604

**Published:** 2022-11-08

**Authors:** Xiaojie Tian, Peng Yi, Fu Zhang, Jinlong Lei, Yiguang Hong

**Affiliations:** 1 Department of Control Science and Engineering, Tongji University, Shanghai 201804, China; 2 Shanghai Research Institute for Intelligent Autonomous Systems, Shanghai 201210, China; 3 Department of Mechanical Engineering, Hong Kong University, Hong Kong 999077, China

**Keywords:** place recognition, loop closure, simultaneous localization and mapping (SLAM), unstructured objects, point cloud segmentation, temporal verification

## Abstract

Place recognition is an essential part of simultaneous localization and mapping (SLAM). LiDAR-based place recognition relies almost exclusively on geometric information. However, geometric information may become unreliable when faced with environments dominated by unstructured objects. In this paper, we explore the role of segmentation for extracting key structured information. We propose STV-SC, a novel segmentation and temporal verification enhanced place recognition method for unstructured environments. It contains a range image-based 3D point segmentation algorithm and a three-stage process to detect a loop. The three-stage method consists of a two-stage candidate loop search process and a one-stage segmentation and temporal verification (STV) process. Our STV process utilizes the time-continuous feature of SLAM to determine whether there is an occasional mismatch. We quantitatively demonstrate that the STV process can trigger false detections caused by unstructured objects and effectively extract structured objects to avoid outliers. Comparison with state-of-art algorithms on public datasets shows that STV-SC can run online and achieve improved performance in unstructured environments (Under the same precision, the recall rate is 1.4∼16% higher than Scan context). Therefore, our algorithm can effectively avoid the mismatching caused by the original algorithm in unstructured environment and improve the environmental adaptability of mobile agents.

## 1. Introduction

As the first step towards the realization of autonomous intelligent systems, simultaneous localization and mapping (SLAM) has attracted much interest and made astonishing progress over the past 30 years [1]. Place recognition or loop closure detection gives SLAM the ability to identify previously observed places, which is critical for back-end pose graph optimization to eliminate accumulated errors and construct globally consistent maps [2,3]. Benefiting from the popularity of cameras and the development of computer vision, vision-based place recognition has been widely studied. However, cameras inevitably struggle to cope with illumination variance, poor light conditions, and view-point change [4]. Compared with camera, LiDAR is robust to such perceptual variance and provides stable loop closures. Thus, LiDAR-based recognition has drawn more attention recently. LiDAR-based place recognition is achieved by encoding descriptors directly from geometric information or segmented objects. Then, similarity is assessed by the distance between descriptors, such as multi-view 2D projection (M2DP) [5], bag of words (BOW) [6], scan context (SC) [7], pointnetvlad [8], and overlapTransformer [9]. Descriptors are extracted from local or global geometric information (3D point clouds). Segmatch [10], semantic graph based place recognition [11], semantic scan context (SSC) [12], and RINet [13] leverage the segmented objects to define descriptors.

**Figure 1:**
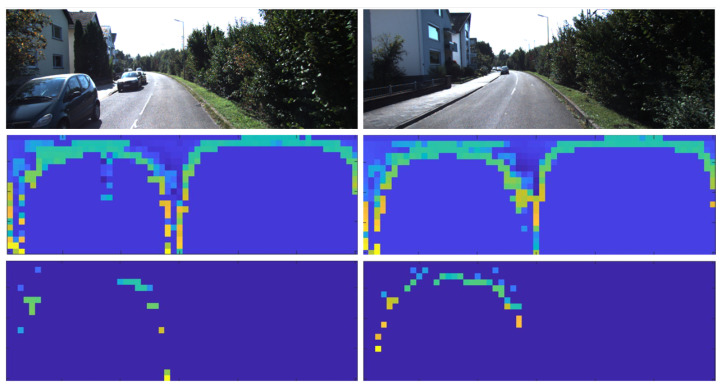
Example of false positive detected by Scan context and triggered by our temporal verification module. Top figures: frame 4058 and 4180 of KITTI sequence 00. The vegetation on the right side makes them difficult to distinguish. Since the ground truth distance between these two frames is 148.64 m, they should not be considered as loop closure. Middle figures: colormap corresponding to scan context before segmentation. Bottom figures: segment scan context of corresponding frame represented by colormap. The left side of colormap indicates the preserved buildings, and the empty right side indicates that the vegetation has been removed. After segmentation, these two frames become distinguishable. If we directly use Scan context, the distance between them is 0.1488, resulting in false positive. Our segment scan context acquires a distance up to 0.327, thus, avoiding outliers.

In this paper, we define relatively large regular objects as structured objects (buildings, ground, trunks, etc.) and others as unstructured objects (vegetation, small moving objects, noise points, etc.). In fact, vegetation is most likely to appear on a large scale and obscure structured information. Thus, we mainly consider unstructured scenes dominated by vegetation. One key issue faced by the above methods is that outliers will occur when two places show similar features due to large scale vegetation. As shown in Figure 1, large scale tree leaves will significantly increase the similarity of different places and reduce the influence of other critical objects in the scene, resulting in similar descriptors between different places. This type of unstructured environment often causes perception aliasing and limits recall rate. Finally, the SLAM system is severely distorted, and the mobile agent cannot perceive the environment correctly. Therefore, designing a place recognition algorithm that is robust in unstructured-dominated environments is of great importance for enhancing the environmental adaptability of autonomous intelligent systems (such as self-driving vehicles and mobile robots) and promoting the development of autonomous driving, field survey, etc.

In [14], segmentation is first proposed to deal with certain conditions, such as forest, and demonstrates potential for removing non-critical information. Inspired by this, here, we intend to enhance scan context with segmentation to make it suitable for unstructured environments. At the same time, considering the time continuity of SLAM and the occasionality of outliers, we use a piecewise thought. Specifically, temporal verification is exploited to candidate loop to decide whether to trigger re-identification module. Thus, reducing the time consumption of the whole system.

In this paper, we present segmentation and temporal verification enhanced scan context (STV-SC). We first design a range image-based segmentation method. Next, we explain why segmented point clouds can differentiate between structured and unstructured objects. Then a three-stage search process is proposed for effective false positives avoidance. The STV process checks temporal consistency to determine whether triggering re-identification module. If triggered, we will segment point clouds and remove unstructured objects of the matching frames. Finally, outliers will be filtered out by the similarity score recomputed by segmented descriptors.

The main contributions of this paper are as follows:

- We propose a range image-based 3D point cloud segmentation method introducing both geometry and intensity constraints for unstructured objects removal.
- An efficient three-stage loop detection algorithm for fast loop candidate search is proposed while leveraging the STV process for perception aliasing rejection.
- Thorough experiments on KITTI dataset [15] show that our method outperforms scan context and other state-of-the-art approaches. The algorithm is also integrated to a SLAM system to verify online place recognition ability.

This paper is structured as follows. Section 2 reviews the related literature of place recognition in both vision and LiDAR manners. Section 3 introduces the 3D point cloud segmentation algorithm proposed, followed by segment scan context and three-stage search algorithm. Then, the experimental test and its discussion are described in Section 4. Finally, a conclusion is made in Section 5.

## 2. Related Works

Depending on the sensing devices used, place recognition can be grouped into vision-based and LiDAR-based methods. Visual place recognition has been well researched and made significant advancement in the past. Generally, visual approaches represent scene features by extracting multiple descriptors, such as Oriented Fast and Rotated BRIEF (ORB) [16] and Scale-Invariant Feature Transforms (SIFT) [17], to construct a dictionary and then leverage bag of words (BOW) [6] model to measure distance between words that belong to different frames. Recently, a learning-based approach has been used for loop detection [18,19]. NetVlad [18] designed a new generalized VLAD layer and implemented it into CNN to achieve end-to-end place recognition. DOOR-SLAM [20] has verified this method in real world SLAM system. However, image representation usually leads to performance degradation when encountering scenes with light illumination and view-point change. To overcome such issues, researchers intended to develop robust visual place recognition methods [21,22,23] to fit change light and season. In spite of this, these methods can only handle certain scenes.

Unlike a camera, LiDAR is robust to environmental changes stated before, while being rotation-invariant. Now, LiDAR-based recognition is still an advanced and challenging problem for laser SLAM systems. LiDAR methods can be further categorized into local descriptors, global descriptors, and learning-based descriptors. Fast point feature histogram (FPFH) [24], keypoint voting [25], and Combination of Bag of Words and Point Feature [6] are state-of-art approaches based on local hand-crafted descriptors. FPFH [24] is coded by calculating key points and their neighbors’ underlying surface properties, such as normal and curvature. Through reordering dataset and caching previously computed values, FPFP can reduce run time and apply to real-time systems. Wang et al. [25] proposed a new 3D regional descriptors based on gestalt features and then certain number of neighbors will be voted by key points to do a similarity score. Bastian et al. [6] used Normal-Aligned Radial Features to build a dictionary for bag of words model and realized robust key points and scene matching.

However, local descriptors rely on the acquisition of key points and the calculation of geometric features around key points, which usually lose a lot of information and lead to false matching. Especially for unstructured outdoor objects (e.g., trees), key points from such objects are unreliable.

In contrast, global descriptors are independent of key points and leverage the global point clouds. Multi-view 2D projection (M2DP) [5] is a novel global descriptor from multi-view 2D mapping of 3D point cloud. This descriptor is designed by the left and right singular vectors of each mapping’s density signature. Giseop et al. [7] divided the 3D space into 2D bins and coded each bin by the maximum height of points in this bin. Then, the global descriptor is represented as a two-dimensional matrix called Scan context. The matching of frames is performed by calculating the cosine distance between scan context in column-wise way. Scan context outperforms existing global descriptors and shows remarkable rotation invariance, which allows it to handle reverse loops. Based on scan context, ref. [26] explored the value of intensity. By integrating both geometry and intensity information, they developed intensity scan context and proved that intensity can reflect information of different objects. Meanwhile, they proposed a binary search process, which reduces the computation time significantly.

In recent years, learning based methods have been proposed gradually. Segmatch [10] first segments different objects from original point clouds and then extracts multiple features from each object, such as eigenvalue and shape histograms. Finally, they utilized a learning-based classifier to matching objects of different scenes. Kong et al. [11] leveraged semantic segmentation to build a connected graph by the center of different objects and used CNN network to match scenes by judging the similarity of graphs. Refs. [12,27] proposed semantic scan context, which encodes each bin by semantic information. However, learning-based method is usually computationally expensive for the training process and cannot adapt to various outdoor environments due to the limitation of training data.

Global descriptors show excellent performance, but still cannot handle ambiguous environment caused by unstructured objects and generate outliers. In this paper, inspired by [14], we utilize segmentation to remove unstructured objects of scenes, but remain global information and key structured objects. Then we apply segmented point clouds to scan context and construct segment scan context, which makes different places more distinguishable and effectively prevents perceptual aliasing.

## 3. Materials and Methods

### 3.1. System Overview

**Figure 2:**
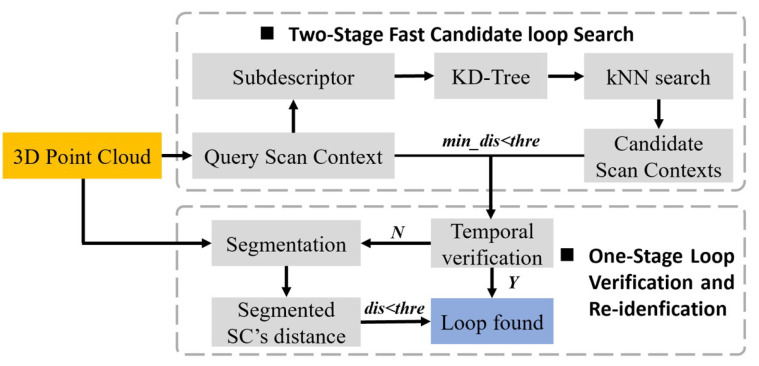
The pipeline of the proposed STV-SC framework. Grey dotted box above: the two-stage fast candidate loop closure search process, including a k-Nearest Neighbor (kNN) search process and a similarity scoring process. Grey dotted box below: our STV process.

An overview of the proposed framework is demonstrated in Figure 2. First, the system acquires original 3D point clouds from LiDAR and codes it into scan context. Then, sub-descriptor is designed and put into KD-Tree, which is an indexed tree data structure used for nearest neighbor search in large-scale high-dimensional data spaces. A fast k-Nearest Neighbor (kNN) search is then implemented to find nearest candidates from KD-Tree. Then, by calculating minimum distance between query scan context and candidate scan contexts, we can tell whether there is a candidate loop closure. If it exists, our STV process is conducted. The temporal verification will determine whether to trigger re-identification procedure. Finally, once the temporal verification is met, we consider it to be a true loop. Otherwise, we will segment the original point cloud and then use the segmented scan context to calculate new distance. The re-identification procedure utilizes this distance to judge whether a loop is found. The detailed description of these modules is given below.

### 3.2. Segmentation

The segmentation module includes two submodules, ground removal and object segmentation. Scan context encodes each bin by taking the maximum height, hence ground points are usually useless and will lead to the increased similarity of different scenes in flat areas. On the other hand, the presence of numerous unstructured objects, such as trees, grass, and other vegetation, will cover the structured information, generating similar descriptors between different places. Meanwhile, it is evident that noises generally do not persist in a certain position over time. Thus, they generally appear scattered and form small-scale objects. Here, we use object segmentation to remove unstructured information in the environment and retain key structured information to prevent mismatches.

We denote each frame of point cloud from the LiDAR as $P=\{p_{1},p_{2},\ldots ,p_{n}\}$. For fast cluster-based segmentation, the 3D point cloud is projected into a $M_{r}\times M_{c}$ 2D range image *R* for point cloud ordering, where

(1) $$M_{r}=\frac{360^{\circ }}{Res_{h}}\;,\;M_{c}=N_{scans}.$$

$Res_{h}$ is the horizontal resolution and $N_{scans}$ is the line number of LiDAR. Each value of the range image is represented by the Euclidean distance from the sensor to the corresponding point cloud in 3D space. Then, we use a column-wise approach for ground point evaluation on the range image like [28], while leveraging intensity for validation.

After removing the ground, we perform a range image-based object segmentation to classify point clouds into distinct clusters, which is based on [29] but with some improvements according to the characteristics of LiDAR. Specifically, we integrate geometry and intensity constraints for clustering. Previous study [30] showed that different objects exhibit different reflected intensity. Since intensity can be obtained directly from LiDAR, it can serve as an additional layer of validation for clustering. We can judge whether two points $p_{a}$ and $p_{b}$ belong to object $O_{k}$ by the following mathematical expression. Meanwhile, we set $\left(a_{1},a_{2}\right)$ and $\left(b_{1},b_{2}\right)$ as their coordinates in the range image, respectively:

$$p_{a},\;p_{b}\in O_{k}$$

$$s.t.\;||a_{1}-b_{1}\left|\right|=1\;or\;||a_{2}-b_{2}\left|\right|=1$$

$$\theta >\epsilon_{g}$$

$$||I\left(p_{a}\right)-I\left(p_{b}\right)\left|\right|<\epsilon_{i}$$

$$\theta =\arctan\frac{d_{2}\sin\gamma }{d_{1}-d_{2}\cos\gamma }$$

(2) I(p)=κ(ψ(p),d).

**Figure 3:**
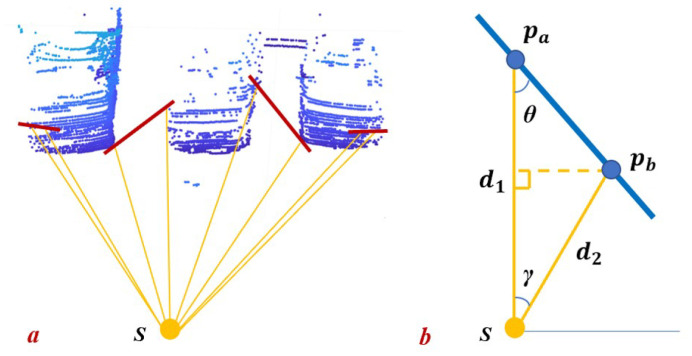
Interpretation of geometry constraint for segmentation. (**a**): three parking cars and laser beams from sensor S. The red line represents the line spawned by two adjacent points. (**b**): geometric abstraction of (**a**). $p_{a}$ and $p_{b}$ represent two adjacent points.

In (2), as shown in Figure 3, *d* stands for the range value from LiDAR to 3D point cloud. θ is the angle between the line spawned by $p_{a},p_{b}$ and the longer one of OA and OB. $\epsilon_{g}$ and $\epsilon_{i}$ are predefined thresholds. Additionally, ψ(p) denotes the intensity of point *p* and κ is an intensity calibration function using distance, which can be obtained by practice.

Noticed that as the first-layer judgment, geometry constraint plays a major role. As the second-layer of validation, intensity prevents objects of different types from being clustered together, i.e., under-segmentation.

Moreover, due to the fixed angle between laser beams, points distributed near the sensor are relatively dense, while points far away are sparse. If a fixed geometric threshold is used, we cannot balance the distant and near points. Specifically, if a large threshold is used, the distant points will be over-segmented, and if a small threshold is used, the nearby points will be under-segmented. Thus, in the near area, using a large $\epsilon_{g}$ can prevent different objects from being grouped together, while using a small $\epsilon_{g}$ in the far area can avoid the same object being segmented into multiple objects.

To achieve more accurate segmentation at different distances, we design a dynamic adjustment strategy. Threshold will be dynamically adjusted as

(3) $$\epsilon_{g}=\epsilon_{g}^{i}-\frac{R(x,y)}{p}q,$$

where *p* denotes step size and *q* is the decay factor. $\epsilon_{g}^{i}$ stands for the initial value of $\epsilon_{g}$.

**Figure 4:**
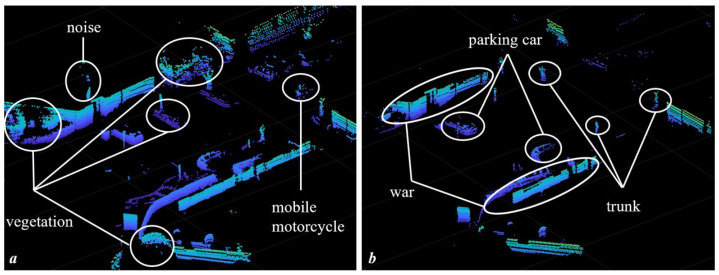
Visualization of the segmentation process. (**a**): original point clouds of one frame. Vegetation, small moving object, and noise are present. (**b**): segmented point clouds, which shows that unstructured vegetation, noise, etc., are removed.

Finally, a breadth-first search based on constraints in (2) is conducted on range image for object clustering. The idea of our segmentation comes from the fact that unstructured objects (mainly vegetation) are filled with gaps, such as leaves. When the laser beams pass through the gaps, the range difference will become large, which will cause large scale vegetation to be separated into small clusters. In the meantime, noise is also a small object. Therefore, we can distinguish structured and unstructured objects by the size of the clusters. In this paper, we treat clusters with more than 30 points or occupying over 5 laser beams as structured objects. As shown in Figure 4, noises, ground, and vegetation are removed, while structured parts, such as buildings and parking cars, are preserved.

### 3.3. Segment Scan Context

**Figure 5:**
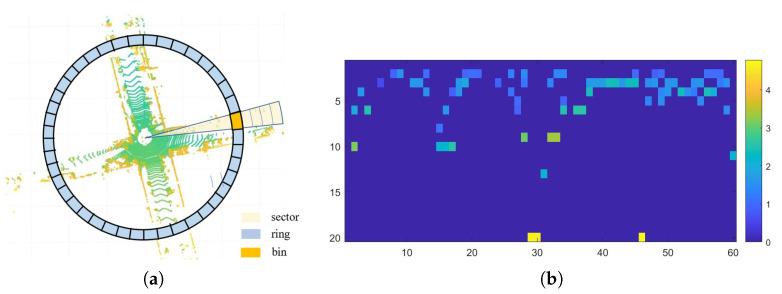
Description of scan context. (**a**): top view of a LiDAR scan, which is separated into bins by rings and sectors. (**b**): colormap of our segment scan context.

Scan context [7] encodes the scene with the maximum height and then represents it by a 2D image. Figure 5a is the top view of original point clouds. Taking the LiDAR as the center, $N_{r}$ rings are equidistantly divided in the radial direction. In the azimuth direction, $N_{s}$ sectors are divided by equal angles. The area where rings and sectors intersect are called bins. For each bin, a unique representation of the maximum height of point clouds within it is used. Therefore, we can project the 3D point clouds into a 2D matrix of $N_{r}\times N_{s}$, called scan context. Let $L_{max}$ represents the maximum sensing range of the LiDAR, then the gaps of rings and sectors are $\frac{L_{max}}{N_{r}}$ and $\frac{2\pi }{N_{s}}$, respectively. By adjusting them, we can set the resolution of scan context.

However, since scan context uses the maximum height as the unique encoding, it usually results in perceptual aliasing when facing large scale unstructured objects. Like trees on both sides of road, they usually have the same height. Therefore, when encountering scenes dominated by unstructured objects, we merely maintain key structured information obtained via point cloud segmentation. Denote point clouds of a segmented LiDAR scan as $P^{seg}$, segment scan context *D* is expressed by

(4) $$D=\left(d_{ij}\right)\in R^{N_{r}\times N_{s}}\;,\;d_{ij}=\phi \left(P_{ij}^{seg}\right).$$

$P_{ij}^{seg}$ are points in a bin with ring index *i* and sector index *j* and ϕ denotes the function to obtain the maximum height of all point clouds in this bin. Particularly, if there is no point in the bin, its value is set to zero. Visualization of our segment scan context is in Figure 5b. After segmentation, descriptors exhibit discrete blocks representing different structured objects.

### 3.4. Three-Stage Search Algorithm

After projecting original point clouds into scan context, the matching process is dedicated to calculating the minimum distance between the descriptor $D_{t}$ obtained at time *t* and the $D=\{D_{1},D_{2},\ldots ,D_{t-1}\}$ stored previously. Then, the distance determines whether there is a loop closure. In order to achieve fast search and effectively prevent mismatches, we design a three-stage search and verification algorithm.

Stage 1: Fast k-Nearest Neighbor search. Obviously, searching in the database directly using scan context will generate numerous decimal operations, which will slow down the search speed. Here, we perform fast search by extracting sub-descriptors. First, scan context is binarized as follows. Let *B* denotes the matrix after binarization:

(5) $$\begin{array}{c} B\left(x,y\right)=\left\{\begin{array}{cc} & \;0,\;\mathrm{if}\;D(x,y)=0,\\ & 1,\;\mathrm{otherwise}. \end{array}\right. \end{array}$$

Then, for each row *r* of *B*, we count the number of non-empty bins by calculating $L_{0}$ norm:

(6) $$\nu \left(r_{i}\right)={∥r_{i}∥}_{0}.$$

Finally, we construct a one-dimensional sub-descriptor $H=(\nu \left(r_{1}\right),\nu \left(r_{2}\right),\ldots ,\nu \left(r_{n}\right))$ that fulfills rotation invariance. By putting *H* into KD-Tree, we can achieve fast kNN search and provide *k* candidates for the next stage.

Stage 2: Similarity score with column shift. This step will directly use the corresponding scan context to find the nearest frame from the candidates obtained in stage 1. Let $D^{q}$ denotes the scan context of query scan. $D^{c}$ denotes one candidate scan context. A column-wise accumulation of cosine distances is used to measure the distance between $D^{q}$ and $D^{c}$. The distance is:

(7) $$\varphi \left(D^{q},D^{c}\right)=\frac{1}{N_{s}}\sum_{i=1}^{N_{s}}\left(\frac{c_{i}^{q}\cdot c_{i}^{c}}{∥c_{i}^{q}∥\cdot ∥c_{i}^{c}∥}\right),$$

where $c_{i}^{q}$ and $c_{i}^{c}$ are the *i*-th column of $D^{q}$ and $D^{c}$, respectively. In practice, mobile agents may revisit one place from different view-points. To achieve rotation invariance, we conduct a column shift process as

(8) $$\varphi_{min}\left(D^{q},D^{c}\right)=\min_{j\in [1,N_{s}]}\varphi \left(D^{q},D_{j}^{c}\right),$$

where $D_{j}^{c}$ means shift $D^{c}$ by *j* columns and $\varphi_{min}$ represents the final smallest value. If $\varphi_{min}$ is lower than the predefined threshold $\epsilon_{l}$, then we obtain a candidate $D^{c}$ for next stage.

Stage 3: Temporal verification and re-identification (STV process). To effectively prevent the generation of false positives, we design a temporal verification module for this candidate loop. Since the detection process of SLAM is continuous in time, the nodes near a true loop also have high similarity. Furthermore, true loops usually exist continuously, while outliers are sporadic. Therefore, we adopt a piecewise idea to verify candidate loop pair:

(9) $$T\left(D_{m},D_{n}\right)=\frac{1}{N_{t}}\sum_{k=1}^{N_{t}}\varphi_{min}\left(D_{m-k},D_{n-k}\right),$$

where $N_{t}$ means the quantity of frames involved for temporal verification. If T less than a threshold $\epsilon_{t}$, we treat it as a true loop. Otherwise, we regard this frame as ambiguous environment and the re-identification module with our segment scan context will be triggered. Specifically, we segment original point clouds and calculate distance between segment scan context of candidate loop pair. Since we have obtained the shift value in the previous stage, we can directly use the result in Equation (8) to calculate the new distance:

(10) $$\varphi_{seg}\left(D^{segq},D^{segc}\right)=\varphi \left(D^{segq},D_{j^{*}}^{segc}\right),$$

where $j^{*}$ represents the shift value when $\varphi_{min}\left(D^{q},D^{c}\right)$ reaches. Finally, if $\varphi_{seg}$ still less than a threshold $\epsilon_{s}$, we group it into inliers; otherwise, we discard it.

Algorithm 1 depicts our search process in detail, where num_diff represents the minimum interval between two frames that can become a loop closure. min_dis means minimum distance.
**Algorithm 1** Tree-stage search process**Require:** 
Original point cloud P of current frame at time *t*.**Require:** 
Scan context $D^{q}$ of current frame at time *t*.**Require:** 
Sub-descriptors of the previous frames in KD-Tree.**Require:** 
Previous scan contexts D stored before *t* 1:k← 50, q← index of current frame. 2:num_diff← 50, min_dis← 100,000. 3:Build the sub-descriptor *H* of the current frame (Equations (5) and (6)) and insert it into KD-Tree. 4:**if**q>k**then** 5:   Find *k* nearest candidates in KD-Tree (kNN search). 6:   **for** i=1 to *k* **do** 7:     ii← frame index of ith candidate. 8:     **if** ii−q>num_diff **then** 9:        Calculate the distance φ between frame *q* and ii (Equations (7) and (8)).  10:        **if** φ<min_dis **then**  11:          min_dis←φ, $D^{c}\leftarrow D^{ii}$.  12:        **end if**  13:     **end if**  14:   **end for**  15:   **if** $min\_dis<\epsilon_{l}$ **then**  16:     Temporal verification of $D^{q}$ and $D^{c}$ (Equation (9).  17:     **if** $\tau <\epsilon_{t}$ **then**  18:        Loop found!  19:     **else**  20:        Segment P to get $P^{seg}$ (Equation (2)).  21:        Construct segment scan context $D^{seg}$ (Equation (4)).  22:         Calculate the distance $\varphi_{seg}$ between $D^{segq}$ and $D^{segc}$ (Equation (7)).  23:        **if** $\varphi_{seg}<\epsilon_{s}$ **then**  24:          Loop found!  25:        **end if**  26:     **end if**  27:   **end if**  28:**end if**

## 4. Experimental Results and Discussion

In this section, we conduct a series of experiments to verify the effectiveness of our STV process for unstructured scenes. Moreover, the discussion regarding each experiment is also presented. The performance of our algorithm is compared with other state-of-art global descriptors. All experiments are performed on a computer equipped with an Intel Core (TM) i5-10210U CPU. To compare with Scan context [7] and test online capability, our algorithm is implemented both in MATLAB and C++.

### 4.1. Experimental Setup

**Figure 6:**
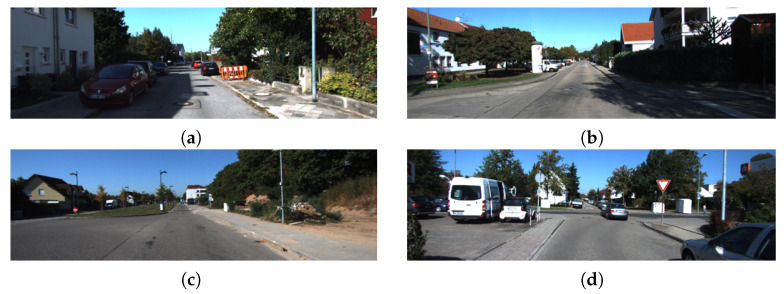
Typical scenes from KITTI sequences. (**a**) sequence 00; (**b**) sequence 05; (**c**) sequence 06; and (**d**) sequence 08. These scenes are dominated by unstructured objects, which can easily cause mismatches.

We select four sequences (00, 05, 06, and 08) from the KITTI dataset [15], all of which contain a large number of typical scenes dominated by unstructured objects (mainly vegetation). As shown in Figure 6, these outdoor scenes provide sufficient experimental resources for our algorithm.

In order to show higher accuracy and exhibit the application value of the algorithm, our parameter settings are similar to scan context-50 [7]. This means that in the first stage we will select 50 nearest neighbors, while ensuring real-time performance. If the ground truth Euclidean distance of matched pair is less than 4m, we consider it to be an inlier. Since $\epsilon_{l}$ and $\epsilon_{t}$ have the same physical meaning, we make them equal in the experiment. Other parameter values used are listed in Table 1.

### 4.2. Statistical Analysis

To illustrate that our STV process can increase the distinguishability in scenes with large scale unstructured objects and effectively avoid the occasional mismatches brought by such scenes. We perform a statistical analysis.

The 4000∼4400th frames of KITTI sequence 00 contain a lot of places dominated by vegetation. Many of these frames are highly susceptible to mismatches, which are discovered through our temporal verification module.

**Figure 7:**
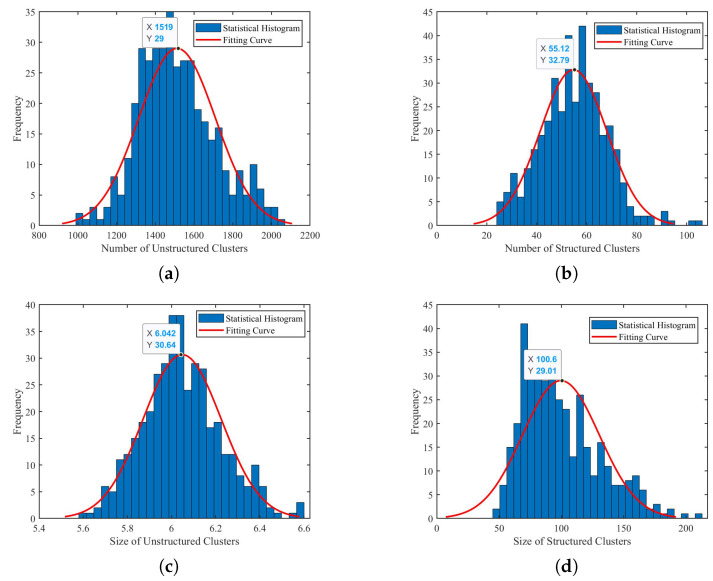
Comparison of segmentation results between structured and unstructured objects. (**a**,**b**): Distribution of the number of unstructured and structured clusters in each frame, respectively. (**c**,**d**): Distribution of the average size of unstructured and structured clusters in each frame, respectively.

We first carry out analysis on the structured and unstructured objects of the selected 400 frames to demonstrate that the segmentation module described in Section 3.2 can indeed separate unstructured objects from structured objects. Figure 7 presents our statistical results. We can find that the clustering number of structured objects after segmentation is much less than that of an unstructured one. The mean values in Figure 7a,b demonstrate a difference of about 30 times. We represent the size of a cluster by the number of points included. Figure 7c,d show that the former tend to be larger clusters, while the latter are small in size due to gaps in vegetation or noises. Generally, structured clusters are more than 10 times larger than unstructured clusters. Therefore, we naturally think of using the size of the cluster to remove vegetation, etc. In subsequent experiments, we will retain clusters with more than 30 points or occupying over 5 laser beams as structured objects.

**Figure 8:**
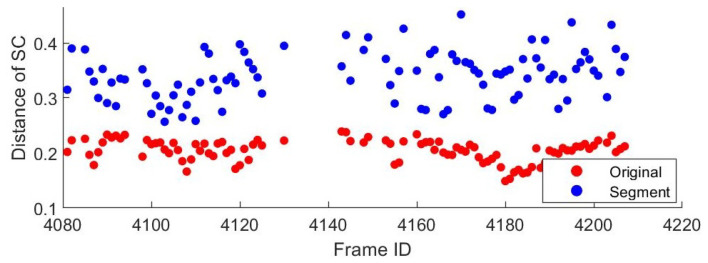
Comparison of false loop pairs’ similarity scores before and after segmentation.

Second, we compare the similarity scores of these 400 pairs of false positives before and after segmentation. As shown in Figure 8, the scores between different places are significantly increased after removing the unstructured objects, as vegetation always has a high degree of similarity. It means improved distinguishability between false loop closures. This allows our algorithm to directly discard mismatches when encountering such places.

### 4.3. Dynamic Threshold Evaluation

In our segmentation algorithm, as the first step judgment, the geometric threshold plays a more critical role in accurate segmentation. According to the characteristics of laser beams, we design a dynamic adjustment strategy of $\epsilon_{g}$, as shown in Equation (3), which can prevent under-segmentation of near objects and over-segmentation of far objects compared with the fixed geometric threshold.

Here, we use the control variable method to test the influence of the dynamic threshold on the experimental results, so as to provide a parameter reference for next experiment. Specifically, we compare the precision and recall rates of fixed and dynamic thresholds with different initial values of $\epsilon_{g}$. Experiments are performed on KITTI sequences 00 and 08, which can provide more convincing references due to their large number of complex and typical unstructured scenes. From the results in Table 2, we can see that under the same initial value, the dynamic threshold tends to achieve higher recall and precision rates than the fixed one. Moreover, we can conclude that the initial value of $\epsilon_{g}$ is best set between 50 and 60.

Therefore, in the following experiments, we set parameters of dynamic threshold as $\epsilon_{g}^{i}=60$, p=10 and q=1.

### 4.4. Precision Recall Evaluation

We leverage precision-recall curves to comprehensively evaluate the performance of our STV-SC method in environments where large scale unstructured objects exist. The performance of our place recognition algorithm is compared with Scan context [7] and M2DP [5], since both are state-of-art global descriptors and neither specifically considers unstructured scenes. In particular, our algorithm is enhanced from Scan context, so the performance comparison with Scan context in unstructured environments is quite important.

**Figure 9:**
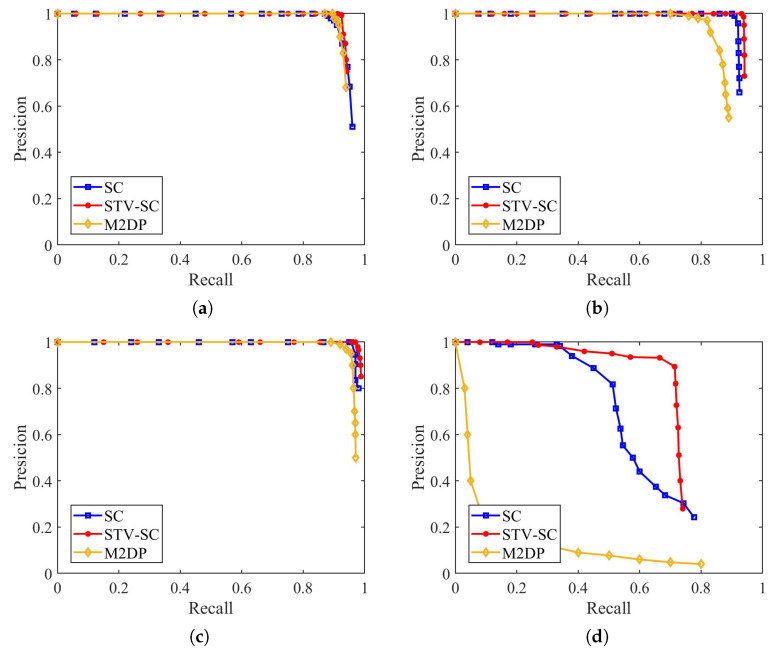
Precision-recall curves on KITTI dataset. (**a**) sequence 00; (**b**) sequence 05; (**c**) sequence 06; and (**d**) sequence 08. The performance of the algorithms is measured by the area enclosed by the curves and the coordinate axes.

As shown in Figure 9, the experiments are conducted on sequences 00, 05, 06, and 08. Since sequence 08 only has reverse loop, it can verify that our algorithm maintains the rotation invariance of Scan context.

Our proposed algorithm outperforms other approaches in all sequences. This is because in the suburban where the roads are surrounded by trees, the geometric information for place recognition is limited. For example, the frames we mentioned in Section 4.2, Scan context will cause mismatches due to the existence of vegetation. However, our method can mitigate the impact of vegetation and avoid many mismatches caused by unstructured objects. That is, under the same recall rate, STV-SC can obtain higher precision rate. As for sequence 08, M2DP performs poorly due to its inability to achieve rotation invariance. However, our algorithm achieves improved performance while maintaining rotation invariance. The residual outliers come from jungles with few or no structured objects or scenes where the structured parts are still very similar so that the geometric information can no longer meet the requirements of place recognition.

In the application, we pay more attention to the recall rate under high precision. Table 3 shows the recall of sequences 00, 05, and 06 at 100% precision. Since sequence 08 is more challenging, we take the recall rate when the precision is 90%. It is obvious that our method outperforms other approaches which do not consider unstructured objects. Compared with the original Scan context, the recall rate of our STV-SC algorithm on different sequences is increased by 1.4% to 16%. In particular, in sequence 08, an environment with a lot of vegetation. Other algorithms often have poor performance, while our algorithm improves the recall rate by more than 15%.

### 4.5. Time-Consumption Analysis

Compared to Scan context, our method adds segmentation and temporal verification (STV) process. Since the re-identification module does not require search and shift actions, the main time consumption of STV is concentrated in the segmentation module. In the meantime, subject to temporal verification, re-identification process is not always triggered, but only used when encountering ambiguous environment. As the main time-consuming module, segmentation uses a range image-based breadth-first search, whose time consumption is fairly small.

**Figure 10:**
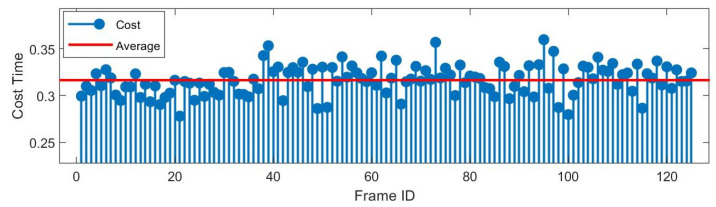
Time-consumption result of 120 triggered frames on KITTI 00. In the case of triggering re-identification, the average time consumption of the whole system is 0.316 s.

Under the same conditions as Scan context-50 [7], we record the place recognition time consumption (cost time of STV-SC) for more than 100 triggered frames in Figure 10. Even at the peak, the time consumption is less than 0.4 s. The average time consumption of these 120 frames is 0.316 s (the original scan context is 0.201 s under 0.2 m${}^{3}$ point cloud downsampling), which is within a reasonable range (2–5 HZ on Matlab).

### 4.6. Online Loop-Closure Performance

Now, we show the online performance of our STV-SC algorithm. Our algorithm is integrated into the well-known LiDAR odometry framework LOAM [31]. Specifically, our method is used as the loop closure detection module of LOAM, then the detected loop is added to the pose graph as an edge. GTSAM [32] is applied for back-end graph optimization. Finally, a drift-free trajectory is obtained. The experiments run on Robot Operating System (ROS Melodic) and perform on KITTI 00.

**Figure 11:**
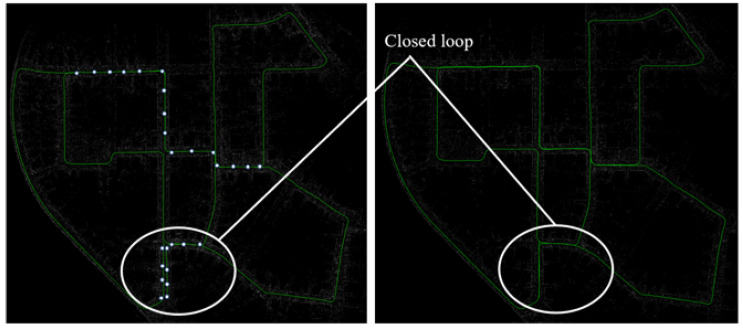
Online loop-closure performance of STV-SC on KITTI 00. Left figure shows the trajectory without loop closure detection and pose graph optimization. The trajectory in the white circle exhibits noticeable drifts. Right figure shows the trajectory after pose graph optimization.

The white dots in Figure 11 represent examples of detected loop closures. As shown in the estimated trajectory, our method can effectively detect loop closures and eliminate drift errors in real time, even in unstructured-dominated environments.

## 5. Conclusions

In this paper, we have proposed STV-SC, a new Scan context-based place recognition method that integrates segmentation and temporal verification process, which gives the original algorithm the ability to handle unstructured environments and enhances the stability of mobile agents in special and complex environments. By summarizing the characteristics of unstructured objects, we design a novel segmentation method to distinguish unstructured and structured objects according to the size of clusters. In addition, for more accurate segmentation we adopt a geometric threshold that varies with range value. In the matching part, we design a three-stage algorithm. Based on the temporal continuity of SLAM system, if temporal verification is not satisfied, the re-identification module will be triggered. Thus, effectively avoiding mismatches caused by unstructured objects. Comprehensive experiments on the KITTI dataset demonstrate that our segmentation method can effectively distinguish different types of objects. STV-SC achieves higher recall and precision rates than Scan context and other state-of-art global descriptors in vegetation-dominated environments. Specifically, it is considered that under the same precision, the recall rate can be improved by 1.4∼16% by our algorithm in different datasets. Meanwhile, the average time consumption of STV-SC is 0.316 s which is within a reasonable bound and proves that the our algorithm can be run in the SLAM system online.

## Figures and Tables

**Table 1:** Parameter List.

Parameter	Value
Maximum radius ($L_{max}$)	80
Number of rings ($M_{r}$)	20
Number of sectors ($M_{s}$)	60
Segmentation threshold ($\epsilon_{g}$)	60
Segmentation threshold ($\epsilon_{i}$)	0.5
Re-identification threshold ($\epsilon_{s}$)	0.2–0.3
Frames of temporal verification ($N_{t}$)	2

**Table 2:** Precision and recall rates of different $\epsilon_{g}$, *p* and *q*.

Parameter			Sequence 00		Sequence 08	
$\epsilon_{g}^{i}$ (°)	p	q	Precision	Recall	Precision	Recall
50	-	-	0.875	0.653	0.998	0.916
55	-	-	0.880	0.707	0.998	0.916
55	20	0.5	0.881	0.711	0.998	0.916
60	-	-	0.894	0.714	0.946	0.912
60	10	1	0.915	0.714	0.998	0.916
65	-	-	0.720	0.714	0.934	0.919
65	10	1	0.809	0.714	0.948	0.918

**Table 3:** Recall at 100% precision on KITTI 00, 05, and 06; Recall at 90% precision on KITTI 08.

	Sequence 00		Sequence 05		Sequence 06		Sequence 08	
Methods	Precision	Recall	Precision	Recall	Precision	Recall	Precision	Recall
Scan Context	1.000	0.870	1.000	0.900	1.000	0.956	0.900	0.550
STV-SC	1.000	0.912	1.000	0.931	1.000	0.970	0.900	0.714
M2DP	1.000	0.896	1.000	0.761	1.000	0.890	0.900	0.020

## Data Availability

Not applicable.

## Abbreviations

The following abbreviations are used in this manuscript:

SLAM	Simultaneous Localization and Mapping
STV	Segmentation and Temporal Verification
SC	Scan Context
LOAM	Lidar Odometry and Mapping

## References

[1] Cadena C., Carlone L., Carrillo H., Latif Y., Scaramuzza D., Neira J., Reid I., Leonard J.J. (2016). Past, Present, and Future of Simultaneous Localization and Mapping: Toward the Robust-Perception Age. IEEE Trans. Robot..

[2] Saeedi S., Trentini M., Seto M., Li H. (2016). Multiple-robot simultaneous localization and mapping: A review. J. Field Robot..

[3] Cattaneo D., Vaghi M., Valada A. (2022). Lcdnet: Deep loop closure detection and point cloud registration for lidar slam. IEEE Trans. Robot..

[4] Taketomi T., Uchiyama H., Ikeda S. (2017). Visual SLAM algorithms: A survey from 2010 to 2016. IPSJ Trans. Comput. Vis. Appl..

[5] He L., Wang X., Zhang H. M2DP: A novel 3D point cloud descriptor and its application in loop closure detection. Proceedings of the 2016 IEEE/RSJ International Conference on Intelligent Robots and Systems (IROS).

[6] Steder B., Ruhnke M., Grzonka S., Burgard W. Place recognition in 3D scans using a combination of bag of words and point feature based relative pose estimation. Proceedings of the 2011 IEEE/RSJ International Conference on Intelligent Robots and Systems.

[7] Kim G., Kim A. Scan context: Egocentric spatial descriptor for place recognition within 3d point cloud map. Proceedings of the 2018 IEEE/RSJ International Conference on Intelligent Robots and Systems (IROS).

[8] Uy M.A., Lee G.H. Pointnetvlad: Deep point cloud based retrieval for large-scale place recognition. Proceedings of the IEEE Conference on Computer Vision and Pattern Recognition.

[9] Ma J., Zhang J., Xu J., Ai R., Gu W., Chen X. (2022). OverlapTransformer: An Efficient and Yaw-Angle-Invariant Transformer Network for LiDAR-Based Place Recognition. IEEE Robot. Autom. Lett..

[10] Dubé R., Dugas D., Stumm E., Nieto J., Siegwart R., Cadena C. Segmatch: Segment based place recognition in 3d point clouds. Proceedings of the 2017 IEEE International Conference on Robotics and Automation (ICRA).

[11] Kong X., Yang X., Zhai G., Zhao X., Zeng X., Wang M., Liu Y., Li W., Wen F. Semantic graph based place recognition for 3d point clouds. Proceedings of the 2020 IEEE/RSJ International Conference on Intelligent Robots and Systems (IROS).

[12] Li L., Kong X., Zhao X., Huang T., Li W., Wen F., Zhang H., Liu Y. SSC: Semantic scan context for large-scale place recognition. Proceedings of the 2021 IEEE/RSJ International Conference on Intelligent Robots and Systems (IROS).

[13] Li L., Kong X., Zhao X., Huang T., Li W., Wen F., Zhang H., Liu Y. (2022). RINet: Efficient 3D Lidar-Based Place Recognition Using Rotation Invariant Neural Network. IEEE Robot. Autom. Lett..

[14] Shan T., Englot B. Lego-loam: Lightweight and ground-optimized lidar odometry and mapping on variable terrain. Proceedings of the 2018 IEEE/RSJ International Conference on Intelligent Robots and Systems (IROS).

[15] Geiger A., Lenz P., Urtasun R. Are we ready for autonomous driving? the kitti vision benchmark suite. Proceedings of the 2012 IEEE Conference on Computer Vision and Pattern Recognition.

[16] Rublee E., Rabaud V., Konolige K., Bradski G. ORB: An efficient alternative to SIFT or SURF. Proceedings of the 2011 International Conference on Computer Vision.

[17] Lowe D.G. Object recognition from local scale-invariant features. Proceedings of the Seventh IEEE International Conference on Computer Vision.

[18] Arandjelovic R., Gronat P., Torii A., Pajdla T., Sivic J. NetVLAD: CNN architecture for weakly supervised place recognition. Proceedings of the IEEE Conference on Computer Vision and Pattern Recognition.

[19] Kendall A., Grimes M., Cipolla R. Posenet: A convolutional network for real-time 6-dof camera relocalization. Proceedings of the IEEE International Conference on Computer Vision.

[20] Lajoie P.-Y., Ramtoula B., Chang Y., Carlone L., Beltrame G. (2020). DOOR-SLAM: Distributed, online, and outlier resilient SLAM for robotic teams. IEEE Robot. Autom. Lett..

[21] Anoosheh A., Sattler T., Timofte R., Pollefeys M., Van Gool L. Night-to-day image translation for retrieval-based localization. Proceedings of the 2019 International Conference on Robotics and Automation (ICRA).

[22] Milford M.J., Wyeth G.F. SeqSLAM: Visual route-based navigation for sunny summer days and stormy winter nights. Proceedings of the 2012 IEEE International Conference on Robotics and Automation.

[23] Arshad S., Kim G.-W. An Appearance and Viewpoint Invariant Visual Place Recognition for Seasonal Changes. Proceedings of the 2020 20th International Conference on Control, Automation and Systems (ICCAS).

[24] Rusu R.B., Blodow N., Beetz M. Fast point feature histograms (FPFH) for 3D registration. Proceedings of the 2009 IEEE International Conference on Robotics and Automation.

[25] Bosse M., Zlot R. Place recognition using keypoint voting in large 3D lidar datasets. Proceedings of the 2013 IEEE International Conference on Robotics and Automation.

[26] Wang H., Wang C., Xie L. Intensity scan context: Coding intensity and geometry relations for loop closure detection. Proceedings of the 2020 IEEE International Conference on Robotics and Automation (ICRA).

[27] Li Y., Su P., Cao M., Chen H., Jiang X., Liu Y. Semantic Scan Context: Global Semantic Descriptor for LiDAR-based Place Recognition. Proceedings of the 2021 IEEE International Conference on Real-time Computing and Robotics (RCAR).

[28] Himmelsbach M., Hundelshausen F.V., Wuensche H.-J. Fast segmentation of 3D point clouds for ground vehicles. Proceedings of the 2010 IEEE Intelligent Vehicles Symposium.

[29] Bogoslavskyi I., Stachniss C. Fast range image-based segmentation of sparse 3D laser scans for online operation. Proceedings of the 2016 IEEE/RSJ International Conference on Intelligent Robots and Systems (IROS).

[30] Kashani A.G., Olsen M.J., Parrish C.E., Wilson N. (2015). A review of LiDAR radiometric processing: From ad hoc intensity correction to rigorous radiometric calibration. Sensors.

[31] Zhang J., Singh S. LOAM: Lidar odometry and mapping in real-time. Proceedings of the Robotics: Science and Systems, University of California.

[32] Kaess M., Johannsson H., Roberts R., Ila V., Leonard J.J., Dellaert F. (2012). iSAM2: Incremental smoothing and mapping using the Bayes tree. Int. J. Robot. Res..

